# Book Reviews

**Published:** 1907-08

**Authors:** 


### BOOK REVIEWS.

THE PRACTICE OF OBSTETRICS.--By American Authors. Edited by Charles Jewett, M. D., Professor of Obstetrics in the Long Island College Hospital, Brooklyn, N. Y. In one handsome octavo volume of 786 pages, with 445 engravings in black and colors and 36 full-page colored plates. Cloth, $5.00 net; leather, $6.00 net; half morocco, $6.50 net. Lea Brothers & Co., New York and Philadelphia.

It is quite a rare occurence in medical literature to find a work of composite authorship appearing in successive new editions. Generally the demand is finished by the first issue. This fact has been the subject of some speculation, the general explanation being that a composite book, perishing in what should be its youth, fails in adaption to its environment in one or both of two essentials: it lacks either the homogeneity or the completeness of a good look by a single author. This defect, however, is individual with the book, and not inherent in the plan. There are indeed, manifest advantages in handling a major subject by collaboration, the most conspicuous being the higher and more uniform level of authority thus attainable. Completeness without duplication is a matter of proper editing. Applying these considerations to a concrete case, it may be inferred that the demand for successive editions of Jewett's Practice of Obstetrics by American Authors indicates a well-edited work of proved high quality. Dr. Jewett and his corps of co-authors have again revised their work thoroughly to the latest date, both in text and in illustrations, in which it has always been notably rich. It is a work equally suited to the needs of obstetrician or student.

---

It is claimed that suicides among children and young persons are increasing in Germany, and that during the last twelve years 1,152 have been reported, and that more than half of them were driven to it by failure in their school examinations or overwork at school.

